# Implementation of a Tunnel System for Scaling-Out High-Quality Cassava Planting Material

**DOI:** 10.3390/plants14192983

**Published:** 2025-09-26

**Authors:** Jazmín Vanessa Pérez-Pazos, Deimer Fuentes-Cassiani, Sol-Mara Regino, Jorge-Luis García, Nilson Osorio, Amaury Espitia, Hernando Araujo, Roosevelt H. Escobar, Amparo Rosero

**Affiliations:** 1 Centro de Investigación Turipaná, Corporación Colombiana de Investigación Agropecuaria—AGROSAVIA, Cereté 250047, Colombia; jvperez@agrosavia.co (J.V.P.-P.); sregino@agrosavia.co (S.-M.R.); jgarciah@agrosavia.co (J.-L.G.); aespitia@agrosavia.co (A.E.); 2 Centro de Investigación Motilonia, Corporación Colombiana de Investigación Agropecuaria—AGROSAVIA, Codazzi 202050, Colombia; dfuentes@agrosavia.co; 3 Centro de Investigación Turipaná, Corporación Colombiana de Investigación Agropecuaria—AGROSAVIA, Carmen de Bolívar 250047, Colombia; ngregorio@agrosavia.co (N.O.); haraujo@agrosavia.co (H.A.); 4 The Americas HUB, Alliance of Bioversity International and CIAT, Km 17, Recta Cali, Palmira 763537, Colombia; r.escobar@cgiar.org; 5 Centro de Investigación Obonuco, Corporación Colombiana de Investigación Agropecuaria—AGROSAVIA, Pasto 520001, Colombia

**Keywords:** cassava seed system, low-cost technology, innovation, scaling-up, TxRP

## Abstract

The production of high-quality cassava planting material is a key strategy for mitigating the spread of pests and diseases. To promote the adoption of such strategies by farmers, it is essential to strengthen local capacities through knowledge transfer and the incorporation of innovative technologies, such as tunnels for rapid propagation (TxRPs), which have been successfully implemented in various international contexts. This study appraised the performance of four industrial cassava (Manihot esculenta Crantz) varieties—Corpoica Tai, Corpoica Belloti, Corpoica Ropain, and Corpoica Sinuana—under tunnel conditions at two locations on the Caribbean coast of Colombia. Planting material consisted of mini-cuttings (7–9 months old) with three buds. Five successive harvest cycles were assessed by measuring key growth parameters, including plant height, leaf number, SPAD (Soil Plant Analysis Development) chlorophyll index, leaf area, and biomass (dry weight and nutrient content). Environmental conditions within the tunnels, such as temperature and humidity, were regulated to promote rapid sprouting and accelerated growth of the cuttings. However, sprouting, vigor, and overall growth performance varied by variety. All four cassava varieties produced high-quality cuttings (>20 mm in diameter and >6 leaves), suitable for further seedling propagation. Cutting vigor increased across cycles, with productivity rising from over 60 cuttings/m^2^ in the first cycle to more than 180 cuttings/m^2^ by the fifth. Substrate mixtures enhanced both physical and chemical soil properties, depending on the source (CRT or CBL). The addition of coco peat or sand effectively minimized environmental impacts by preventing substrate compaction. The findings demonstrate the potential of tunnel-based systems to accelerate the production of high-quality cassava planting material, supporting improved productivity and sustainability in cassava cultivation for both farmers and industry stakeholders.

## 1. Introduction

Cassava (*Manihot esculenta* Crantz) is a staple food in many cultures, with the capacity to promote local employment and income generation for numerous farming communities in developing countries, particularly in Africa, Asia, and Latin America [1]. Historically regarded as a subsistence crop and a food security reserve during times of scarcity, cassava is undergoing a rapid transformation into a cash crop. Nowadays, it is used not only as a raw material for flour and starch production (and its derivatives), but also as beneficial livestock feed in the major producing countries [2,3]. Regarding cassava production, in 2023 the countries reporting the highest global production were Nigeria, the Democratic Republic of Congo, Thailand, and Ghana, each with yields exceeding 25 million tons (MT). Among South American countries, Brazil was the leading cassava producer with 18 MT, followed by Paraguay, Peru, and Colombia https://www.fao.org/faostat/es/#data/QCL (accessed on 25 May 2025). In Colombia, cassava production in 2023 was reported at 1 MT, led by the Caribbean region—particularly the areas of Bolívar, Córdoba, Sucre, and Magdalena—which each produced over 0.22 MT https://www.agronet.gov.co/estadistica/Paginas/home.aspx?cod=1 (accessed om 25 May 2025).

Major phytosanitary problems in cassava are linked to its vegetative propagation system, which enables the accumulation and transmission of diseases across growing cycles. Worldwide, key diseases such as Cassava Wishes’ Broom Disease (CWBD) [4], Cassava Frog-Skin Disease (CFSD) [5], Cassava Mosaic Disease (CMD) [6], Cassava Brown Streak Disease (CBSD) [7], and Cassava Bacterial Blight (CBB) [8] are among the most damaging and are mainly disseminated through infected cuttings [9,10]. As cassava is a vegetatively propagated crop, systemic infections could persist and intensify over successive generations, reducing plant vigor, yield, and seed quality [11,12]. Some diseases may remain asymptomatic during the early stages of infection, for example CFSD, which limits cassava production in Latin America, including Colombia; its pathology is characterized by corky, fissured root symptoms that reduce the starch content and marketability of roots, and infected stems consequently allow the spread of the disease [5]. The causal agent of CFSD had long remained elusive, with previous associations pointing to phytoplasma and reovirids. However, Jiménez et al. [13] demonstrated through field studies and high-throughput sequencing that single infections caused by Torrado viruses—specifically Cassava Torrado-like Virus (CsTLV)—are sufficient to induce CFSD symptoms without the involvement of any other pathogen. In this context, there are many difficulties in detecting and eliminating virus-infected cuttings in informal seed systems with limited diagnostic capacity. Therefore, there is an essential requirement of disease-free planting material to mitigate the spread of systemic diseases in cassava-producing areas and their effects on yield and quality losses [14]. These constraints underscore the importance of implementing a cassava clean seed system of improved varieties and landraces and integrated disease management approaches to safeguard its production.

Seed system governance is structured through both formal and informal rules, involving key stakeholders such as farmers, Non-Governmental Organizations (NGOs), National Agricultural Research System (NARS), and seeds custodians. In the case of cassava, the informal seed system predominates, with farmers acquiring planting material through collect, exchange, donations, or purchase from other producers [15,16]. In Colombia, the production of certified cassava seed is regulated by Colombian Agricultural Institute (ICA) under Resolution N° 00015141 but this practice is not so frequent. Although the release of new improved varieties requires the delivery of certified material, the limited capacity impacts their supply and adoption. The traditional seed production system, based on the use of conventional stem cuttings, faces significant challenges, including limited availability of planting material in both quantity and quality by producers during a specific period for planting.

One of the main constraints of cassava production systems is the low/lack of access to high-quality planting material (HQPM) from improved varieties and landraces [11,17]. Poor storage methods are also problematic, given that cassava stems are living and metabolically active tissue particularly sensitive to dehydration and unable to withstand prolonged exposure to high temperatures and low relative humidity that affect seed quality [18]. Cassava has low multiplication rates, potential phytosanitary problems spread through stem cuttings, and based on seed quality it can show low sprouting rates that reduce the effective plant density per unit area which can affect its yield [19].

Cassava is propagated by mature stems [20], with multiplication rates around 10–20 cuttings per mature plant after one year of previous culture [21]. However, research institutions have developed rapid multiplication techniques for producing HQPM, such as tissue culture [22], shoot induction methods, the use of mini-cuttings in cages or plastic bags [19], and rapid cassava seed multiplication in tunnels, developed by CIAT [23].

Cassava is so important for food security and income generation for small-scale farmers in the north-coast of Colombia; however, the low availability of planting material, both in quantity and quality, is a major constraint faced by this production system. Although the current ICA resolution regulates the production of certified cassava seed in Colombia, the traditional system based on the use of stem cuttings from the previous crop cycle does not comply with it. Therefore, the incorporation of a tunnel system for the speed of HQPM offers a potential solution to this issue. This study aims to evaluate and monitor the response of four preferred industrial cassava varieties under TxRPs to produce an appropriated technology that can supply small-scale farmers with planting material on-farm and contribute to improving their crop system.

## 2. Materials and Methods

### 2.1. Locations

The TxRPs were built in Cereté (CRT) and Carmen de Bolívar (CBL), two locations with dry and humid conditions, respectively, in the Caribbean region of Colombia (Table 1). The experiment was conducted in a cycle in 2024.

### 2.2. Origin of Plant Material and Selection of Mini-Cuttings

The plant material was produced in accordance with ICA Resolution No. 00015141 to produce certified seed in Colombia, starting from in vitro plants and continuing through to the harvest of mature stems for use at the certified seed level. Four industrial cassava varieties were used for this test: Corpoica Tai (Tai 8, common name Tai), Corpoica Belloti (SM 2775-4, common name Belloti), Corpoica Ropain (GM 273-57, common name Ropain) and Corpoica Sinuana (SM 1411-5, common name Sinuana). From a field plot of certified planting material, healthy stems from each variety were selected. Plants with mechanical damage were eliminated from this study. Later, stems were cut using a handsaw to obtain three buds each. Disinfection was carried out by immersing them in a solution with Metalaxyl-M (4%) and Mancozeb (64%) (2 g·L^−1^) and copper oxychloride (2 g·L^−1^) in combination for five minutes, followed by shade drying. Elements for personal protection including goggles, plastic gloves, plastic boots, aprons, and a mask respirator were used in this phase. For each variety, internode distance (cm), length (cm), and diameter (cm) were recorded from 15 mini-cuttings per variety.

### 2.3. Description of the Multiplication Tunnels

#### 2.3.1. Structure of the Tunnels for Rapid Propagation

**Figure 1:**
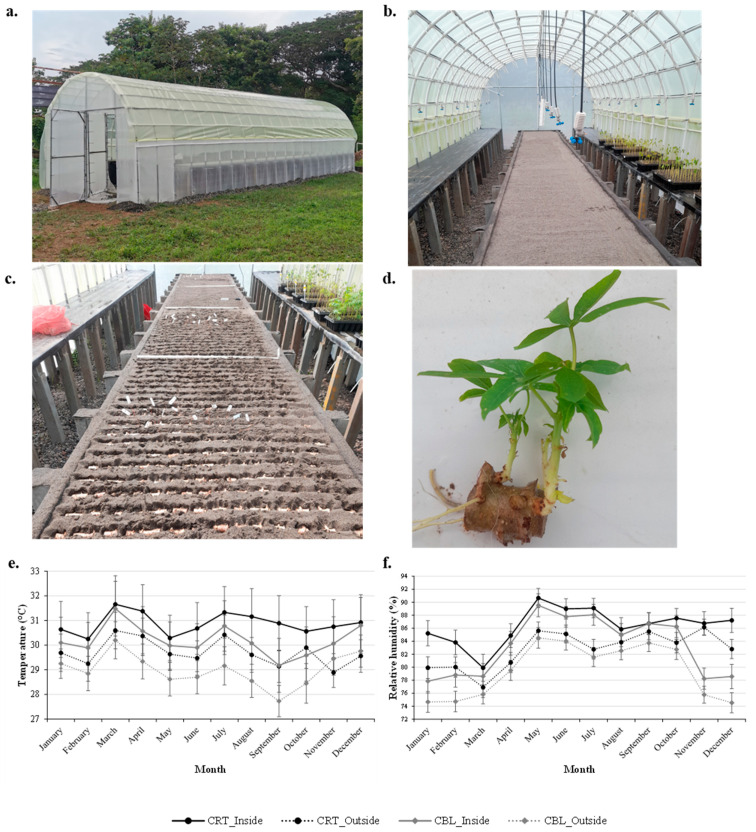
(**a**) External view of tunnels built in each location: 9 m in length, 3.5 m in width, and 2.4 m in height. (**b**) Internal distribution of each tunnel showing walking area between central beds (that receive mini-cuttings) and two lateral flat beds that receive seeds trays with harvested sprouts by each cycle. (**c**) Two-to-three-node cuttings planted on the central sand bed. The density/m^2^ of mini-cuttings is variety-dependent. (**d**) Details of sprouting for each bud/mini-cutting under the tunnel system. (**e**,**f**) Diagrams of average temperature and relative humidity values comparing conditions inside and outside of the propagation tunnel. CRT: Cereté; CBL: Carmen de Bolívar. Error bars represent standard errors.

A TxRP is a simple structure designed to speed the sprouting of all outgrowth points present on each mini-cutting. The tunnel dimensions in this study were approximately 6–9 m length, 3.5 m width, and 2.4 m height. The tunnel was covered with clear plastic to allow sunlight entry and maintain temperature and humidity inside (gauge 8; light transmission: 80–85%; light diffusion: 50–55%). The structure was made from PVC or galvanized metal. The system enhances the multiplication rate by providing high temperature and humidity and sunlight conditions, which are crucial for the rapid and continuous sprouting of buds [23] (Figure 1). Inside it was built a central bed that uses washed medium river sand as a substrate to grow 2–3-node cuttings, and 2 flat tables at each side that each receive trays with harvested 45 days old sprouts from the central beds. All areas inside are subject to the fogging system, which consists of three lines (one above each bed), allowing an environment with controlled high humidity and high temperature to speed sprouting time and growing rates in all explants. In the Colombian Caribbean, mist irrigation intervals of 5 min every 3 h during early plant growth are extended to 8–10 min as foliage increases, ensuring substrate moisture levels of 9–11% to mitigate water stress [23]. Among the materials that can be used to build the central bed are bamboo, mats, wood, plastic wood, metal, boxes made with car tents, and wire among others.

Temperature and relative humidity were recorded using Aranet temperature and humidity sensors, with one sensor placed at the center of the tunnel at a height of 2 m above the ground and another located in an external area. Data were logged every 5 min.

#### 2.3.2. Central Bed and Content

The central bed has a length of 8 m and 1 m width, with a base layer of medium-sized stones serving as a filter. The central bed is filled with 100% sand, considering the availability of sand [23] in the CRT and CBL locations. Before placing the sand in the central bed, it was disinfected by solarization for at least 3 days. To achieve this, it must be watered well, disposed in a block with 7–10 cm height, and covered with clear plastic in an open area to achieve full sun incidence for at least 3 days. This is used to control most weeds and some pathogens.

A chemical analysis of sand in each region was conducted in accordance with the NTC ISO/IEC 17025 standard [24], using the following protocols: pH (active acidity/pH in soils, GA-R-46, version 06, 25 October 2021); available phosphorus by the Bray II method (GA-R-48, version 07, 25 October 2021); electrical conductivity in soils (NTC 5596:2008 Method B); exchangeable cations—calcium, magnesium, potassium, and sodium (GA-R-50, version 09, 25 October 2021); and micronutrients (iron, manganese, copper, and zinc) extracted using a modified Olsen method (NTC 5526:2007 Method D). Organic carbon content was determined following GA-R-119 (version 04, 25 October 2021). In addition, a physical analysis was performed to assess aggregate stability using the Yoder method, as described by Le Bissonnais (2016) [25]. Based on the data obtained, the weighted mean diameter (WMD) and the distribution of fine (<0.5 mm), medium (0.5–2 mm), and coarse (>2 mm) aggregates were calculated following the methodology reported by Pérez-Pazos et al. [26].

#### 2.3.3. Irrigation System and Frequency

To maintain high humidity inside the tunnel, fogging cycle irrigation was applied every 3 h for 5 min during the first 20 days after planting (DAP) which corresponds to the initial growth stage. After 20 DAP, the irrigation cycle was increased to 8–10 min every 3 h to maintain moisture levels in the sand, which support plant establishment.

### 2.4. Establishment of Mini-Cuttings and Management

Mini-cuttings, of each variety in each location, with 2–3 nodes were planted horizontally, 1 cm apart from one another and with 8 cm between rows, at a depth of 3 cm in the central bed of the tunnel. This ensures uniformity in the experiments; a total number of 350 mini-cuttings per variety were planted occupying an area of 2 square meters of the central bed. Monitoring every 8 days through visual inspection was implemented to control and record the occurrence of problems (pests and diseases) in the tunnel, in three planting rows per clone (excluding border rows). Although sprouting efficiency mainly depends on the quality and maturity level of the mature stem used as planting material in the tunnel, a nitrogen-based fertilizer was applied fifteen days after the first harvest to promote optimal sprouting conditions. A total of 200 g of urea (46% nitrogen) was evenly distributed across the central bed to stimulate the development of new shoots. This practice was necessary due to the sandy texture of the substrate, which, although easier to handle because of its loose structure, contains low reserves of nutrients available for root absorption.

### 2.5. Cuttings’ Harvest for Multiplication and Evaluation

The sprout harvest was performed at approximately 40–45 DAP, when most sprouts had reached four to five nodes. During harvesting time, a minimum of one node was left on the original mini-cuttings, and the harvested cuttings had more than threenodes. For the selected cuttings, the following physiological parameters were measured: height, number of leaves, stem diameter, chlorophyll content (SPAD units), and leaf area. For the leaf area measurement, a photo record was taken by cutting all the leaves from the cutting and arranging them fully extended on a white background next to a measuring ruler and with proper labeling. The fresh weight of stems and leaves was recorded too, after which the material was dried in an oven to determine dry weight. After collecting, the remaining cuttings of each variety were harvested and immediately placed in a container with tap water to control sap exudation and prevent dehydration until the transplanting phase. A total of five cuts were carried out throughout the entire growth cycle in the multiplication test, and for each cutting’s harvest, the previously mentioned variables were recorded. Finally, once the growth variables were determined for each cut, all the cuttings planted by variety were collected and counted to estimate the number of cuttings produced per unit area. Since each variety was planted in a two-square-meter plot, the total count was divided by two to estimate the production of cuttings per square meter.

### 2.6. Planting and Monitoring Cuttings in Germination Trays

The sprouts harvested were transplanted into seed trays and tested with four substrate mixtures as follows: S1—soil/sand/vermicompost at 85:10:5%; S2—soil/vermicompost/sand at 60:30:10%; S3—soil/vermicompost/coco peat/sand at 20:20:20:40%; S4—soil 100% as a control set. For each substrate type by region, a chemical analysis was conducted in accordance with the NTC ISO/IEC 17025 standard.

Once planted, the seed trays with harvest sprouts were placed on the flat beds of the tunnel, where they received an irrigation cycle under the same schedule used for the central bed. After 12–15 days in the tunnel, the seed trays were transferred to a shaded area (under 65% shade mesh), where they were irrigated daily or as needed according to weather conditions and substrate moisture to prevent fungi and algae development.

Monitoring through visual inspection of the cuttings was conducted at 8, 15, and 30 DAP to control the appearance of problems caused by pests and diseases. Once the plants reached 30 DAP, the survival percentage (%) for each substrate evaluated was determined. To determine the survival percentage, the total number of cuttings planted (cp) in the germination trays was recorded. After 30 DAP, the number of cuttings that remained alive (ca) in the trays was counted, and survival was calculated as % survival = (ca × 100)/cp.

### 2.7. Statistical Analysis

To assess the behavior of the evaluated variables in cassava cuttings produced from mini-cuttings in propagation tunnels, a principal component analysis (PCA) was performed. The first PCA used location as the classification criterion to identify differences between sites, while a second PCA was conducted using the interaction between harvests and varieties as the classification criterion to determine the variables associated with this interaction. For the variables plant height, number of leaves, stem diameter, and SPAD units, a one-way analysis of variance (ANOVA) was performed to determine statistical differences. Tukey’s Honestly Significant Difference (HSD) test was used for post hoc comparisons at a 95% confidence level. To evaluate the production of cuttings/m^2^ in the multiplication tunnel, an independent analysis of variance was performed for each of the determined factors, as well as paired interactions between factors.

All statistical analyses were conducted using R software version 12.1 [27]. ANOVA was performed using the “onewaytest” package [28], and Tukey’s HSD test was conducted with the “TukeyC” package. PCA was executed using the “princomp” function [29], the PCA biplot was generated using the “fviz_pca_biplot” function, and correlation plots were created using the “corrplot” package [30].

## 3. Results and Discussion

### 3.1. Internal Temperature Conditions of Multiplication Tunnels

The tunnels built in each region proved to be a structurally stable system that, under the agroecological conditions tested, withstood strong winds and heavy rainfall, demonstrating the resilience of this type of infrastructure with relatively low investment (Figure 1a). Internally, the distribution of the central sprouting bed and the lateral pre-adaptation beds had appropriate dimensions for efficient handling and ensured adequate working space and movement for staff (Figure 1b). During implementation, it was observed that the number of mini-cuttings per square meter depends on the cassava clone used (Figure 1c), as discussed in detail below. In all cases, sprout emergence was vigorous and consistent (Figure 1d), indicating a good seed source, maturity of the material, and a favorable environment for early growth. A distinct microenvironment was established within the tunnel when compared to external ambient conditions across all evaluation regions (Figure 1e,f). The increases in both temperature and relative humidity inside the tunnel were statistically significant. Seasonal variation was evident: The highest internal and external temperatures were recorded during March and July, while the lowest values occurred in May and September. Relative humidity (RH) inside the tunnel was lowest in March, coinciding with the regional dry season.

In contrast, the subsequent months showed a progressive increase in humidity, peaking in May. To mitigate excessive internal temperatures and maintain optimal humidity levels, mist irrigation systems were employed. This approach proved effective in promoting sprout emergence and the subsequent growth and development of cassava cuttings. Daily inspections were conducted to ensure proper functioning of the misting nozzles, which are susceptible to clogging and can compromise uniform water distribution. These results were consistent with well-documented responses: the external ambient temperature was always lower than the inside temperature, mainly due to the solar radiation entering the greenhouse through transparent plastic and being trapped there [31]. Similarly, internal humidity responds to outside weather conditions—such as external temperature, external humidity, global radiation, and wind speed—as well as control mechanisms such as ventilation [32]. The internal environmental conditions were previously optimized to support adequate plant growth, with a focus on enhancing production efficiency in controlled environments through climate regulation strategies [33]. In line with earlier findings, cassava varieties exhibit differential responses to cumulative precipitation, temperature, and soil composition, emphasizing the need for variety-specific adaptation strategies [33]. Temperature and RH significantly influence both soil temperature and plant physiological responses. For instance, optimal sprouting in certain cassava varieties has been reported at 28.5–30 °C, while the temperature range supporting maximum photosynthetic activity lies between 25 and 35 °C. Moreover, RH plays a crucial role in regulating stomatal conductance (gs), which controls CO_2_ uptake and transpiration; thus, high RH levels (78–95%) have been associated with enhanced photosynthetic performance in cassava due to improved stomatal function and reduced water stress [34,35].

Regarding the condition of the plant material established in the central beds of the multiplication tunnels, it was found that distance between stem nodes, cutting length, and diameter were slightly different among varieties. Thus, the measurement of mini-cuttings’ characteristics for each variety indicated that Ropain and Sinuana exhibited a greater cutting length (>4 cm) compared to the Belloti and Tai varieties. These results are correlated with internode length, which was also greater (>1 cm) in Ropain and Sinuana (Table 2). Regarding the average mini-cutting diameter, Ropain showed the lowest value (2.06 cm), while Belloti, Sinuana, and Tai had an average diameter ranging from 2.19 cm to 2.22 cm (Table 2).

### 3.2. Characteristics of the Sand Used for the Establishment of the Multiplication Tunnels

The chemical analysis of the sand used in the propagation tunnels according to their locations showed that CBL samples have higher contents of P, S, Ca, Mg, Fe, and Zn compared to those of CRT sand (Table 3).

Regarding the physical analyses of aggregate stability performed on the sands, the results indicate that in both cases the mean weighted diameter was below 0,5 mm (Table 4), a value typical of substrates without structure, such as sand [36]. Concerning aggregate content, a high percentage of fine aggregates was observed in both locations (Table 4), which leads to compaction and reduced aeration, affecting negatively root development [37]. In the case of TxRPs, the use of mini-cuttings allows the sprouting of buds and their development to be less dependent on nutrient availability and sand characteristics, relying primarily on the quality of the mini-cutting source and maturity status of the stem for their growth. However, using sand contributes few nutrients but offers suitable porosity to enhance sprout development. One recommendation is to use medium-textured sand rather than fine sand to improve aeration and to avoid compaction of substrate during growing cycles.

Overall, it was observed that the sand from CRT exhibits a lower mean aggregate diameter and a higher content of fine aggregates compared to the sand used in CBL (Table 3), suggesting that the sand employed in second locations has a better structure than the first one.

### 3.3. Multifactorial Effect of Location, Cut, and Variety on Growth Variables Under TxRPs

The analysis of variance showed that individual factors—location, cut, and variety—as well as their interactions were significant for most of the evaluated variables, with the exception of stem diameter, for which the cut × variety interaction was not significant (Table 5). For all variables, the cut factor showed a major contribution to the model. For the variables plant height, number of leaves, and leaf area, the mean square values indicated a greater contribution from the main effects of cut, location, and variety. Among the interactions, the contributions followed a descending order: location × cut, location × variety, cut × variety, and the triple interaction location × cut × variety. The variables stem diameter, SPAD, FWL (fresh weight of leaves), FWS (fresh weight of stems), and FWT (total fresh weight) showed a greater contribution from the main effects of cut and variety. The location factor contributed more strongly through its interactions, particularly in Location × Cut, Location × Variety, and even in the triple interaction (Table S1). The high effect of locations is associated with the annual cycle of air temperature and other environmental conditions that show heterogeneity among spatial distribution, mainly due to the geographical position, orography, proximity to the sea, and humidity contributions of the continental environments [38], which directly affect the TxRP internal conditions. In the other hand, the differences among cuts could be related to the vigor and nutritional characteristics of the mother stem, which subsequently will affect the survival and rooting of the produced cuttings [39].

**Figure 2:**
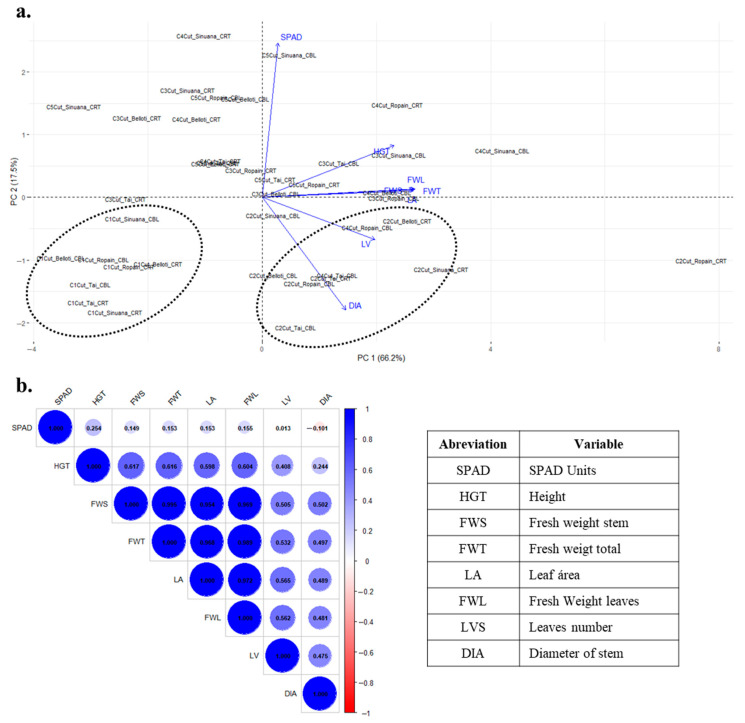
Principal component analysis of four cassava varieties (Belloti, Ropain, Sinuana, and Tai) grown in TxRPs in two localities (CBL and CRT) at different cut times (C1Cut–C5Cut). (**a**) Principal component plot. (**b**) Correlation plot and variable description.

Principal component analysis (PCA) was applied to reduce the dimensionality of the dataset and to explore underlying patterns and groupings among the factors and measured variables; thus, the first two principal components explained 83.7% of the total variance in the dataset (Figure 2a). In line with the analysis of variance, the cuts contributed to a potential grouping, enabling the distinction of cuts 1 and 2, whereas the remaining cuts did not exhibit a clear or consistent pattern. Consistently, the vigor of the mother stem changes or decreases along the successive cuttings and, although additional nutrients are incorporated, the characteristics of the cuttings differ after the second. This phenomenon could be explained due to the vigor and nutritional characteristics of the mother stem defining the vigor of new sprouts [39]. A strong relationship among the variables LA, FWL, FWS, and FWT was observed (Figure 2b). At a functional level, this relationship may be explained by the fact that leaf area is associated with light absorption capacity, which directly influences biomass accumulation in plants [40]. Furthermore, changes in leaf area linked to morphological variation have been reported to alter energy balance within the leaf, thereby regulating biomass distribution between the leaf itself and the supporting and photosynthetic tissues [41].

Regarding the indirect chlorophyll content measured as SPAD units, a negative correlation was observed with the diameter of the stem (DIA) and a very low correlation with the number of leaves (Figure 2b). This may be explained by the fact that good stem development increases the number of leaves, thereby raising the probability of shading, which in turn reduces the availability of light energy for chlorophyll synthesis. Similar behavior has been reported in maize, where leaves exposed to low-light conditions showed increased respiration, which was associated with reduced photosynthetic performance [42]. No relationship was observed between SPAD values and other growth variables (LA, HGT, and FW), suggesting that under tunnel conditions, structural vigor (size/biomass) is not solely related to chlorophyll content but may instead be associated with other physiological process, vigor, and nutritional status of the mini-cuttings [43].

### 3.4. Growth Behavior of Cassava Variety Under TxRPs in Two Locations

**Figure 3:**
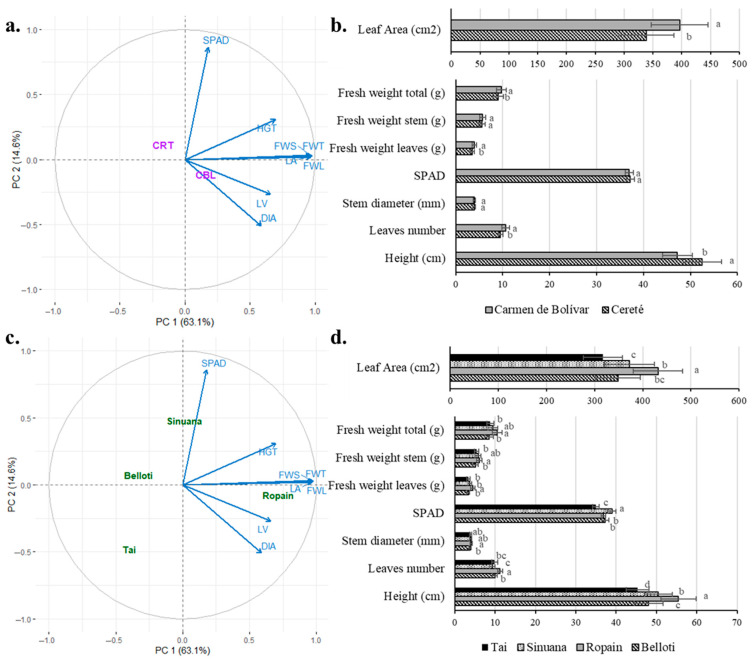
Growth variables of cassava plants established in TxRPs in two locations (CRT and CBL) for four varieties (Belloti, Sinuana, Ropain, and Tai). (**a**) PCA of the overall results obtained from growth variables for the two locations. (**b**) Response of growth variables in the two locations. (**c**) PCA of the overall results obtained from growth variables for variety. (**d**) Response of growth variables of the varieties. Error bars represent standard errors. Different letters denote significant differences according to Tukey’s test (*p* < 0.01).

To further investigate the impact of environmental conditions and varietal performance, PCA analysis was performed; the first two components explained 79.2% of the total variability (PC1: 63.1%; PC2: 14.6%), which is sufficient for analysis as they capture the greatest possible variability with the fewest components. In Figure 3a, CBL appears to be linked to vegetative traits such as diameter and number of leaves, while CRT is located toward the center of the graph, indicating that it is not clearly related to any group of variables. These results suggest that the CBL locality offers more favorable conditions for growth responses. This is consistent with findings of Mbise et al. [12], who reported that cultivation environments significantly impact seedling height and the number of leaves produced. Regarding the response of growth variables in the two locations (Figure 3b), CRT exhibited higher average values for plant height, compared to CBL. In contrast, CBL showed higher values for leaf number, fresh weight, and leaf area than CRT. These findings corroborate the results presented in the PCA that show different effects on growth variables.

The distribution of varieties among the PCA biplot showed differences among them in terms of growth variables (Figure 3c): while Ropain was closer to the leaf area and fresh weight variables, the other varieties were located in different quadrants. This response was clearly confirmed by media comparison analysis (Figure 3d), which showed that Ropain exhibited higher values of height, leaf number, stem diameter, fresh weight, and leaf area. This aligns with observations by Mbise et al. [12] and López [44], who reported that the number of shoots from stem cuttings also depends on the apical dominance characteristic of each variety. As apical dominance strengthens, only the upper shoot develops into the main stem. Additionally, the overall condition of the mother stem—particularly the axillary buds and vigor—had significant effects on the number of nodes, percent sprouting, and number of sprouts [45].

### 3.5. Growth Behavior of Cassava Variety and Cuts Under TxRPs

**Figure 4:**
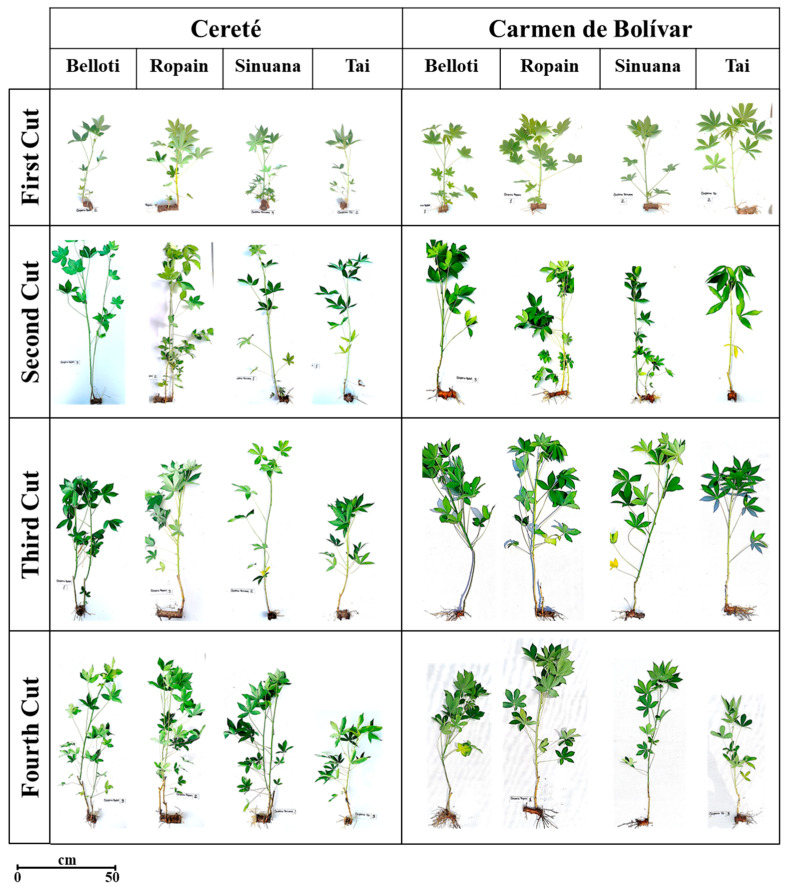
Visual record of cuttings harvested from TxRPs by variety and at two locations (CRT and CBL).

**Figure 5:**
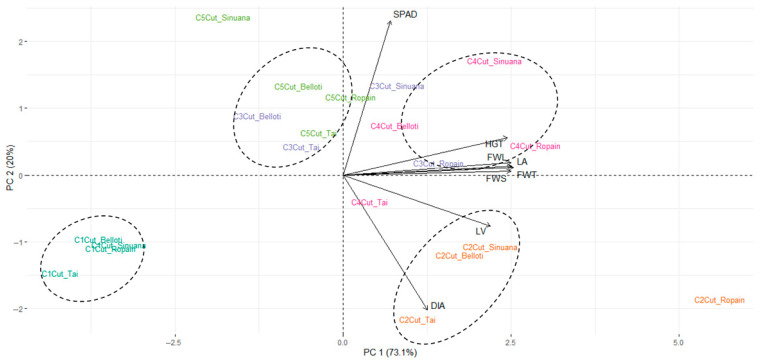
Principal component analysis of four cassava varieties (Belloti, Ropain, Sinuana, and Tai) grown in TxRPs at different cuts. Different colours represent different cuts.

Differences in plant growth among cuts and varieties was observed (Figure 4). These changes were related to general vigor, height, and the other variables of plant growth; however, in all cases the plants showed adequate quality as a planting material. To gain a deeper insight into the differences between varieties and cuts, a principal component analysis (PCA) was performed (Figure 5).

The results of PCA analysis showed the presence of four distinct data groups (Figure 5). The first group corresponds to cassava cuttings obtained from the first cut of the varieties Belloti, Sinuana, Ropain, and Tai, which clustered toward the lower left of the plot and showed no association with any growth variables. The data corresponding to plants from all varieties obtained at the second cut clustered at the lower right of the PCA plot, associated with the variables number of leaves and stem diameter. A cluster corresponding to the fourth cut with the varieties Belloti, Sinuana, and Ropain, as well as the third cut of Ropain, was associated with the variables of height, leaf area, and fresh weights of leaves and stems. Finally, a cluster associated with the SPAD variable corresponds to cuttings from all varieties obtained at the fifth and third cuts, except for Ropain at the third cut (Figure 5).

**Figure 6:**
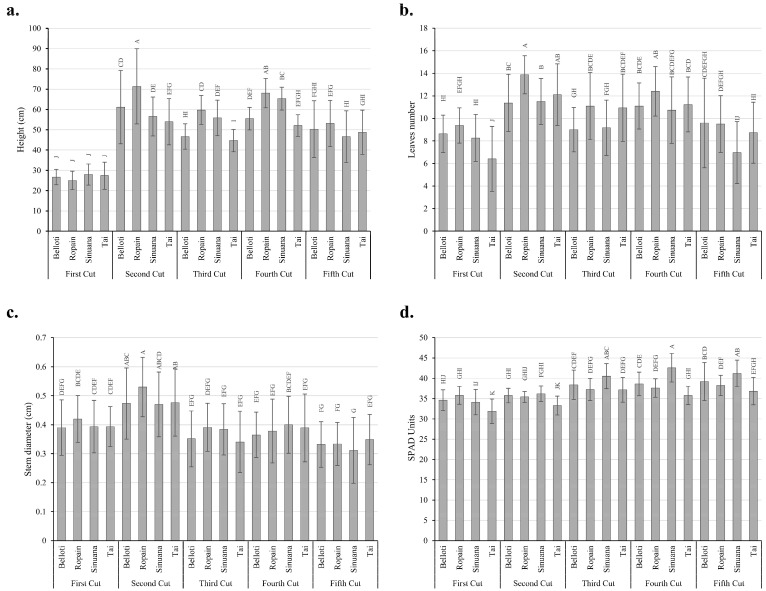
Average growth parameter response of four cassava varieties (Belloti, Ropain, Sinuana, and Tai) grown in TxRPs at different cuts. (**a**) Cutting height. (**b**) Number of leaves. (**c**) Stem diameter. (**d**) SPAD units. Error bars represent standard errors. Different letters denote significant differences according to Tukey’s test (*p* < 0.01).

The relations between cuts and varieties on growth variables were consistent with the responses observed in the media comparison. According to plant height (Figure 6a) and number of leaves (Figure 6b), values at the first cut were significantly lower compared to those at subsequent cuts. Although mini-cuttings can produce functional cuttings during the first month of growth, their vigor is lower compared to those generated in later cuts. Cuttings of Ropain and Sinuana obtained at the fourth harvest exhibited heights ranging from 60 to 75 cm, representing the highest range recorded across all harvests. Although the average height of Belloti and Ropain cuttings at the second harvest exceeded 60 cm, their data showed much greater variability. Edet [46] reported increased starch accumulation in stem cuttings converted into glucose due to high demand from the sprouting system, which impacts sprout yield, leaf area index, and plant height during the early growth stages of cassava. Ropain variety exhibited the highest number of leaves and height. These results are comparable to those reported by Mbise et al. [12], who found a significant difference in the number of cassava leaves produced under low tunnels, where the highest number of leaves recorded was 20 for the evaluated variety, and the lowest was 5. Furthermore, the planting method significantly influenced the number of leaves, with cuttings planted horizontally producing more leaves.

Regarding stem diameter, among cuttings obtained at the second cut, Ropain showed the highest value at 0.5 cm (Figure 6c). In general, after the second cut, there was a process of producing cuttings with greater vigor in terms of stem diameter and number of leaves, which may be related to the availability of nutrient reserves following the first harvest. These results suggest that the mini-cuttings’ capacity to produce cuttings of good size may be achieved by the fourth harvest. These cuttings could have a high potential for bud production, consistent with findings by Meibuko et al. [45] who reported a positive correlation between bud emission capacity and vegetative growth. The minimum average height reached at the fourth harvest in the multiplication tunnels was 52 cm, exceeding the height reported by Mbise et al. [12], who found that the seedling height of cassava varieties was significantly affected by planting methods and cutting size in TxRPs and nurseries, with height ranges between 9 and 17 cm, being higher for cuttings with four nodes. It has also been reported that cassava plant height is strongly positively correlated with leaf area, fresh root weight, and dry matter yield. Similarly, branching in cassava increases leaf production, which in turn enhances light interception, photosynthesis, and consequently yield and its components.

These findings demonstrate the successful process of rapid multiplication of planting material, since previous studies showed that cuttings between 20 and 25 mm in diameter and 6 to 8 visible leaves should be used to produce cassava seedlings with the rapid multiplication method [39] (. Differences among genotypes may be attributed to genetic variation and adaptability to the tunnel environmental conditions [47]. 

The greater height observed in Ropain may be attributed to genetic variation and its adaptability to the tunnel environmental conditions [47]. Additionally, differences in the growth of cassava cuttings may be related to their size, as larger-diameter cuttings contain greater nutrient reserves than smaller ones; thus, newly emerged shoots provide sufficient nutrients necessary for plant growth [48]. These results indicate that, for this dataset, SPAD units best describe their behavior. Regarding SPAD values (Figure 6d), it can be observed that in the third and fifth cut cycle, all varieties recorded average values above 36. SPAD units are related to the relative chlorophyll content; Dey et al. [49] reported that when the relative chlorophyll content in leaves exceeds 35, plants are considered to have a good nitrogen status, which is essential for vegetative growth. Based on these results, it can be inferred that during the third and fifth harvest round, the photosynthetic activity of the plants may be enhanced.

### 3.6. Cutting Yield Estimation and Survival

**Figure 7:**
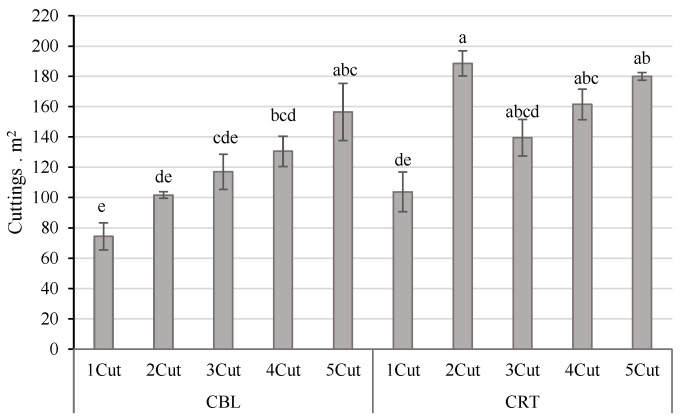
Cutting production/m^2^ obtained in TxRPs in the different cuttings made to from established plant material in the localities Carmen de Bolivar (CBL) and Cereté (CRT). Error bars represent standard errors. Different letters denote significant differences according to Tukey’s test (*p* < 0.01).

To assess cutting production, an analysis of variance (ANOVA) was conducted for each factor. The results indicated no significant differences among the evaluated cassava varieties (*p* = 0.4128); however, significant effects were observed for location (*p* = 0.0014) and cutting number (*p* = 0.0003). Among the interaction terms, only the location × cutting interaction showed a significant effect (*p* = 0.0266), whereas the interactions of location × variety (*p* = 0.6441) and cutting × variety (*p* = 0.9889) were not significant. Regarding the significant location × cutting interaction (Figure 7), a progressive increase in cutting production was observed in the CBL location across successive cuttings. A similar trend was noted in CRT; however, a marked increase was detected at the second cutting, reaching a maximum of 190 cuttings per square meter. Although no differences were observed in cutting production among the evaluated varieties, it is noteworthy that the production range was 124–150 cuttings/m^2^.

**Figure 8:**
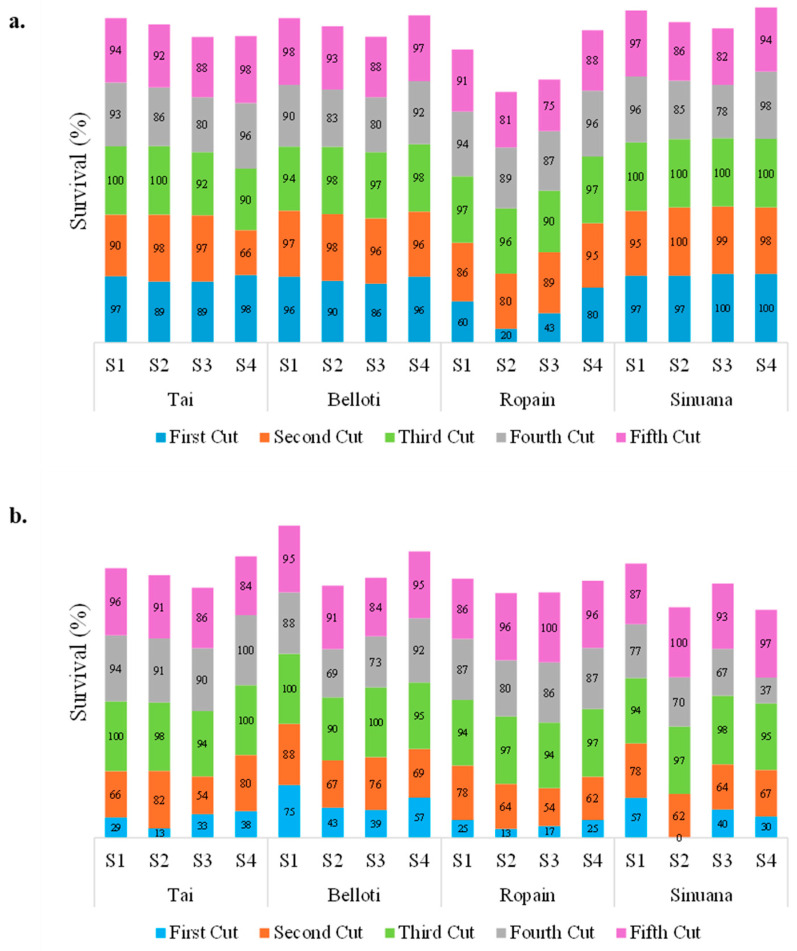
Survival percentage of harvest sprout, 30 DAP on different substrates and locations: (**a**) Cereté location and (**b**) Carmen de Bolivar location. S1: soil/sand/vermicompost at 85:10:5%; S2: soil/vermicompost/sand at 60:30:10%; S3: soil/vermicompost/coco peat/sand at 20:20:20:40%; and S4: soil 100%.

The survival percentage of sprouting harvested/cuttings obtained from TxRPs varied among variety, locations, and cuts (Figure 8). It can be observed that the survival percentages in CRT were, on average, higher than those observed in CBL, with a general average of 92.2% and 78%, respectively. In CRT, for the Tai, Belloti, and Sinuana varieties, values greater than 85% were presented in almost all cuttings and substrates, except for Tai for the second harvest cycle in S4 (soil 100%), where it presented the only value well below this range at 66%. In contrast, Ropain showed a lower survival percentage, especially in the first cutting, with an average of 51%. Substrates S2 and S3 were where they presented the lowest values at 20% and 43%, respectively. However, these percentages improved progressively in subsequent cuts. Regarding the substrates for this location, S4 (soil 100%) tends to show very stable survival results across all cuts for all varieties, with values above 90% for most combinations. In CBL, a drastic reduction in survival values was observed for most variety–substrate combinations across different cuts, with the lowest values generally occurring in the first cut. Ropain and Tai were the varieties with the lowest averages at 20% and 28%, respectively. From the third to the fifth cut, varieties’ survival values across different substrate types tended to increase, with values above 70%. Analyzing substrate behavior, S1 for Belloti tended to show the highest and most stable survival values. These findings are consistent with those of Meibuko et al. [45], who reported significant clonal effects on node number, sprouting percentage, and shoot number. Another important factor is substrate temperature, which directly affects cassava shoot emergence. According to Keating and Evenson [34], soil temperatures below 17 °C reduce sprouting rates, while temperatures close to 30 °C accelerate them. Furthermore, Meibuko et al. [45] mention that the use of coco peat and peatmoss substrates generally led to earlier and more successful germination. Overall, all substrates show good fertility and nutrient availability for seedlings (Table 6); the only element found in the low range is copper in the CRT locality. The pH of the substrates is in the range from slightly alkaline (6.96) to moderate alkaline (7.85), which is favorable for the development of the crop. In both locations, the available phosphorus and potassium found for the substrates was evaluated in the medium-to-high range except for S4 in the locality of CRT where the low range was presented. It is worth noting that the survival variable shows the best results in S1, which presents nutritional characteristics for the plant in the medium-to-high range in most of the elements. In contrast, in CRT most of the substrates presented optimal nutritional characteristics for the development of the cuttings.

## 4. Conclusions

Climatic variables such as temperature and humidity inside tunnels can be effectively modulated, particularly with the use of fogging irrigation systems, to create optimal conditions for rapid sprouting and accelerated growth of cassava cuttings. However, sprouting, vigor, and overall growth rates remain dependent on the variety. All cassava varieties assessed in this study exhibited strong performance, making them suitable candidates for large-scale multiplication strategies using tunnel-based systems. High-quality cuttings (>20 mm in diameter and >6 leaves) were consistently produced for seedling propagation, and cutting vigor increased progressively across cycles, with production rising from over 60 cuttings/m^2^ to more than 180 cuttings/m^2^ by the fifth cycle.

Substrate mixtures improved the physical and chemical properties of the soil, with variations in quality depending on their origin (CRT or CBL). The inclusion of local by-products such as vermicompost, coco peat, and sand reduced the soil compaction, promoting better rooting. This also offers potential for local entrepreneurship through the use of available materials.

This study underscores the potential of tunnel-based systems to expedite the propagation of high-quality cassava planting material, improving the speed of propagation, reducing sprouting time, and ultimately providing superior planting material for both farmers and industry stakeholders.

## Figures and Tables

**Table 1:** Climate characterization of localities used in this study.

Location	Landscape	Climate	Geographic Location	Mean Temperature (°C)	Accumulated Precipitation (mm/Year)
CRT	Plain	Warm humid	8°50′27.47″ N, 75°48′27.56″ W	31.3	1244
CBL	Mountains and Piedmont	Warm dry	9°42′50.8″ N, 75°6′26.9″ W	27.5	1150

**Table 2:** Architectural parameters of mini-cuttings of cassava varieties grown in propagation tunnels.

Variety	Internode Length (cm)	Cutting Length (cm)	Cutting Diameter (cm)
Belloti	0.89 ± 0.30	3.48 ± 0.58	2.19 ± 0.13
Ropain	1.19 ± 0.50	4.39 ± 1.28	2.06 ± 0.21
Sinuana	1.08 ± 0.45	4.11 ± 1.06	2.22 ± 0.35
Tai	0.70 ± 0.31	3.22 ± 0.82	2.21 ± 0.27

**Table 3:** Sand chemical characterization according to their origin (CBL and CRT).

Parameter	Units	CBL Origin		CRT Origin	
		Value	Interpretation	Value	Interpretation
pH		7.99	Alkaline	7.07	Near neutral or neutral
Electrical Conductivity	dS·m^−1^	0.68	Non-saline	0.07	Non-saline
Organic Carbon	g·100 g^−1^	<0.22	Not applicable	<0.22	Not applicable
Organic Matter	g·100 g^−1^	<0.38	Low	<0.38	Low
Available Phosphorus	mg·kg^−1^	30.46	Medium	3.39	Low
Available Sulfur	mg·kg^−1^	19	Medium	4.25	Low
ECEC *	cmol(+)·kg^−1^	6.07	Low	3.09	Low
Available Boron	mg·kg^−1^	0.1	Low	0.1	Low
Acidity	cmol(+)·kg^−1^	ND	Not indicated	ND	Not indicated
Exchangeable Aluminum	cmol(+)·kg^−1^	ND	No restriction	ND	No restriction
Available Calcium	cmol(+)·kg^−1^	4.15	Medium	2.2	Low
Available Magnesium	cmol(+)·kg^−1^	1.73	Medium	0.77	Low
Available Potassium	cmol(+)·kg^−1^	0.12	Low	<0.09	Low
Available Sodium	cmol(+)·kg^−1^	<0.14	Normal	<0.14	Normal
Available Iron	mg·kg^−1^	69.13	High	5.34	Low
Available Cupper	mg·kg^−1^	<1	Low	<1	Low
Available Manganese	mg·kg^−1^	1.66	Low	2.85	Low
Available Zinc	mg·kg^−1^	2.86	Medium	<1	Low
Calcium Saturation	%	68	High	71	High
Magnesium Saturation	%	29	High	25	Medium
Potassium Saturation	%	2	Medium	2	Medium
Sodium Saturation	%	1	Normal	2	Normal
Aluminum Saturation	%	0	Normal	0	Normal

* ECEC: effective cation exchange capacity; dS = decisiemens; cmol = centimol; m = meter; g = gram; mg = milligram; kg = kilogram.

**Table 4:** Sand index characterization and structural stability according to its origin.

Parameter	CBL	CRT
Mean weight diameter (mm)	0.306	0.268
Fine aggregates < 0.5 mm (%)	86.49	94.1
Medium aggregates 0.5 mm–2 mm (%)	13.47	5.9
Extreme aggregates > 2 mm (%)	0.04	0

**Table 5:** Mean square values from the analysis of variance of location, cut, and variety on cassava growth parameters under TxRPs.

SV	df	Height	Leaf Number	Stem Diameter	Leaf Area	SPAD	FWL	FWS	FWT
Model	39	3650.27 ***	86.43 ***	0.08 ***	335,254.68 ***	138.43 ***	31.28 ***	67.05 ***	184.97 ***
Locality	1	4197.61 ***	191.54 ***	0.0017	503,107.37 ***	6.94	37.97 ***	6.43	75.65 *
Cut	4	22,911.61 ***	352.49 ***	0.41 ***	1,502,381.11 ***	591.43 ***	103.28 ***	245.19 ***	662.6 ***
Variety	3	2765.31 ***	99.34 ***	0.02 *	350,419.7 ***	395.69 ***	40.45 ***	24.45 **	123.05 ***
Locality*Cut	4	5467.37 ***	190.72 ***	0.17 ***	682,176.21 ***	238.03 ***	77.33 ***	232.61 ***	574.21 ***
Locality*Variety	3	1443.72 ***	16.4 **	0.03 *	308,907.85 ***	38.58 ***	45.22 ***	71.44 ***	228.3 ***
Cut*Variety	12	650.46 ***	21.51 ***	0.01	78,572.22 ***	39.93 ***	6.91 ***	10.17 *	32.4 **
Locality*Cut*Variety	12	351.2 ***	33.43 ***	0.03 ***	76,062.2 **	24.32 ***	9.98 ***	23.97 ***	62.35 ***
Error	560	39.02	4.07	0.01	27,552.1	6.06	2.03	4.8	13.03

SV: source of variation; df: degree of freedom; FWL: fresh weight leaves; FWS: fresh weight stem; FWT: fresh weight total. Significance levels: * *p* < 0.05; ** *p* < 0.01; *** *p* < 0.001.

**Table 6:** Chemical characterization of the substrates used for the cuttings establishment in seed trays at different locations (CRT and CBL).

Parameter	CRT								CBL							
	S1		S2		S3		S4		S1		S2		S3		S4	
	Val	Analysis	Val	Analysis	Val	Analysis	Val	Analysis	Val	Analysis	Val	Analysis	Val	Analysis	Val	Analysis
pH	7.65	Alk	7.32	Nt	6.96	Nt	7.85	Alk	7.71	Alk	7.18	Nt	7.1	N	7.87	Alk
Electrical Conductivity	2.81	SS	6.55	MS	4.35	MS	1.99	NS	1.62	NS	5.93	MS	4.6	MS	0.59	NS
Organic Carbon	0.27	NA	0.55	NA	1.26	NA	0.25	NA	1.33	NA	2.37	NA	2.42	NA	1.11	NA
Organic Matter	0.47	L	0.95	L	2.17	M	0.43	L	2.29	M	4.09	H	4.17	H	1.91	L
Available Phosphorus	36.88	M	164.19	H	190.2	H	9.81	L	84.73	H	218.33	H	194.62	H	26.9	M
Available Sulfur	139.62	H	234.14	H	124.24	H	137.37	H	40.07	H	152.43	H	116.31	H	19.52	M
ECEC	17.8	M	26.85	H	13.83	M	17.1	M	41.39	H	44.19	H	23.54	H	43.37	H
Available Boron	0.3	M	0.69	H	0.65	H	0.19	L	0.76	H	1.07	H	0.76	H	0.58	H
Available Calcium	8.97	H	13.51	H	7.38	H	8.27	H	32.76	H	30.13	H	14.06	H	35.17	H
Available Magnesium	7.14	H	9.22	H	3.81	H	7.34	H	6.25	H	8.37	H	5.45	H	6.61	H
Available Potassium	0.34	M	2.35	H	1.89	H	0.12	L	1.81	H	4.61	H	3.2	H	1.1	H
Available Sodium	1.35	H	1.77	H	0.75	N	1.37	H	0.57	N	1.08	H	0.83	N	0.49	N
Available Iron	18.37	L	65.88	H	58.38	H	8.21	L	19.39	L	38.94	M	39.08	M	10.53	L
Available Cupper	<1	L	<1	L	<1	L	<1	L	3.31	H	2.71	M	<1	L	4.12	H
Available Manganese	<1	L	2.34	L	6.05	M	<1	L	<1	L	1.54	L	2.86	L	<1	L
Available Zinc	<1	L	3.79	H	3.18	H	<1	L	<1	L	3.09	H	2.47	M	<1	L
Calcium Saturation	50	M	50	M	53	H	48	M	79	H	68	H	60	H	81	H
Magnesium Saturation	40	H	34	H	28	H	43	H	15	M	19	M	23	M	15	M
Potassium Saturation	2	M	9	H	14	H	1	L	4	H	10	H	14	H	3	M
Sodium Saturation	8	N	7	N	5	N	8	N	1	N	2	N	4	N	1	N

ECEC: Effective Cation Exchange Capacity; Val: Value; Analysis: Interpretation of the value; Alk: alkaline; Nt: neutral; SS: slightly saline; MS: moderately saline; NS: non-saline; NA: not applicable; L: low; M: medium; H: high; N: normal. S1: soil/sand/vermicompost at 85:10:5%; S2: soil/vermicompost/sand at 60:30:10%; S3: soil/vermicompost/coco peat/sand at 20:20:20:40%; and S4: soil 100%.

## Data Availability

All the relevant data are available in the manuscript itself. Additional data can be provided on request.

## Supplementary Materials

The following supporting information can be downloaded at https://www.mdpi.com/article/10.3390/plants14192983/s1, Table S1: Mean values of growth variables according to triple interaction Locality x cut x variety
