# Supplementary material for: Implementation of a Tunnel System for Scaling-Out High-Quality Cassava Planting Material
Source: Plants (Basel). 2025 Sep 26;14(19):2983. doi: 10.3390/plants14192983 (PMC12525939; doi:10.3390/plants14192983)
Supplement: Supplementary file 1 [file plants-14-02983-s001.zip › plants-3844270-supplementary.pdf]

Table S1. Mean values of growth variables according to triple interaction Locality x cut x variety

LOCALITY	CUT	VARIETY	Height (cm)		Leaves Number		Stem Diameter (cm)		Leaf Area (cm2)		SPAD		Fres weight leaves (g)		Fres weight stem (g)		Fres weight total (g)	
			Media	Tt	Media	Tt	Media	Tt	Media	Tt	Media	Tt	Media	Tt	Media	Tt	Media	Tt
CBL	1Cut	Belloti	24,93	op	7,47	mnopq	0,330	hijk	137,0	n	32,71	lmn	2,100	hij	2,700	lm	4,800	kl
CBL	1Cut	Ropain	21,67	p	8,93	jklmnopq	0,390	defghij	209,4	jklmn	35,05	ijklm	2,530	ghij	2,710	lm	5,240	jkl
CBL	1Cut	Sinuana	30,40	op	7,00	pqr	0,370	efghijk	269,5	fghijklmn	35,78	ghijklm	2,540	ghij	3,970	ijklm	6,510	fghijkl
CBL	1Cut	Tai	32,93	no	4,13	r	0,390	defghij	173,0	lmn	31,24	n	2,350	hij	3,790	ijklm	6,150	hijkl
CBL	2Cut	Belloti	44,20	klm	13,27	abcde	0,430	bcdefghi	420,9	cdefghijk	34,68	jklmn	3,490	cdefghij	4,980	defghijklm	8,470	defghijkl
CBL	2Cut	Ropain	53,60	ghij	12,80	abcdefg	0,500	abcd	499,0	bcdef	35,62	hijklm	3,680	cdefghij	4,680	fghijklm	8,360	defghijkl
CBL	2Cut	Sinuana	48,40	jklm	12,47	abcdefgh	0,410	bcdefghi	443,8	bcdefghij	37,50	cdefghij	3,380	defghij	4,920	defghijklm	8,300	defghijkl
CBL	2Cut	Tai	43,60	lm	13,87	ab	0,500	abcd	340,7	cdefghijklmn	33,89	klmn	3,730	cdefghij	5,670	cdefghijklm	9,410	defghijkl
CBL	3Cut	Belloti	50,47	ijkl	10,27	fghijklmn	0,380	defghij	416,6	cdefghijk	36,65	efghijk	3,950	cdefgh	5,760	cdefghijkl	9,720	defghijk
CBL	3Cut	Ropain	63,93	cdef	13,73	abc	0,380	defghij	512,9	bcde	36,19	ghijkl	5,300	bcde	7,150	bcdefgh	12,440	bcde
CBL	3Cut	Sinuana	62,80	def	11,20	bcdefghij	0,410	bcdefghi	524,6	bcd	39,23	bcdefg	5,310	bcd	7,880	bcd	13,190	bcd
CBL	3Cut	Tai	48,80	ijklm	13,60	abcd	0,280	jk	483,8	bcdefgh	36,54	fghijk	4,780	bcdef	7,250	bcdefgh	12,020	bcde
CBL	4Cut	Belloti	53,40	ghij	10,93	cdefghijk	0,410	bcdefghi	540,6	bc	37,20	defghijk	5,360	bcd	7,990	bcd	13,350	bcd
CBL	4Cut	Ropain	63,73	cdef	12,93	abcdef	0,440	abcdefgh	464,1	bcdefghi	36,19	ghijkl	4,770	bcdef	6,550	cdefghi	11,320	bcdefg
CBL	4Cut	Sinuana	68,87	cd	12,80	abcdefg	0,470	abcde	661,6	ab	41,00	abc	6,670	b	8,730	bc	15,410	b
CBL	4Cut	Tai	48,00	jklm	12,53	abcdefgh	0,460	abcdef	436,1	bcdefghij	34,99	ijklm	4,630	cdef	6,460	cdefghij	11,080	bcdefgh
CBL	5Cut	Belloti	40,33	mn	11,00	bcdefghijk	0,330	ghijk	344,5	cdefghijklmn	42,22	ab	4,010	cdefgh	5,330	defghijklm	9,330	defghijkl
CBL	5Cut	Ropain	52,80	hijk	8,27	klmnopq	0,310	ijk	347,2	cdefghijklmn	40,00	bcdef	3,340	defghij	5,110	defghijklm	8,450	defghijkl
CBL	5Cut	Sinuana	50,13	ijkl	7,40	nopq	0,370	efghijk	389,5	cdefghijklm	44,14	a	4,060	cdefgh	7,450	bcdef	11,520	bcdef
CBL	5Cut	Tai	40,93	mn	8,67	jklmnopq	0,340	fghijk	311,7	cdefghijklmn	37,80	cdefghij	3,660	cdefghij	5,510	defghijklm	9,170	defghijkl
CRT	1Cut	Belloti	28,47	op	9,80	hijklmnop	0,450	abcdefg	249,8	hijklmn	36,55	efghijk	2,190	hij	4,140	hijklm	6,330	ghijkl
CRT	1Cut	Ropain	28,47	op	9,80	hijklmnop	0,450	abcdefg	146,6	n	36,55	efghijk	2,370	hij	3,240	klm	5,620	ijkl
CRT	1Cut	Sinuana	25,53	op	9,53	ijklmnop	0,410	bcdefghi	189,0	klmn	32,53	mn	2,060	hij	3,420	jklm	5,480	jkl
CRT	1Cut	Tai	21,73	p	8,67	jklmnopq	0,390	defghij	154,3	mn	32,55	mn	1,880	ij	3,360	jklm	5,240	jkl
CRT	2Cut	Belloti	78,13	b	9,47	ijklmnop	0,520	abc	528,5	bcd	36,86	defghijk	4,460	cdefg	8,010	bcd	12,470	bcde
CRT	2Cut	Ropain	89,20	a	14,93	a	0,560	a	804,0	a	35,26	ijklm	8,760	a	12,540	a	21,300	a
CRT	2Cut	Sinuana	64,73	cde	10,53	efghijkl	0,530	ab	491,4	bcdefg	34,94	ijklm	5,230	bcde	9,750	ab	14,980	bc
CRT	2Cut	Tai	64,33	cdef	10,33	fghijklm	0,450	abcdefg	406,4	cdefghijkl	32,70	lmn	3,850	cdefghij	7,300	bcdefg	11,140	bcdefgh
CRT	3Cut	Belloti	42,80	lm	7,73	lmnopq	0,330	hijk	246,7	ijklmn	40,18	bcd	2,380	hij	3,700	ijklm	6,080	hijkl
CRT	3Cut	Ropain	55,53	fghij	8,47	jklmnopq	0,410	bcdefghi	343,7	cdefghijklmn	38,29	cdefghi	3,910	cdefgh	5,070	defghijklm	8,970	defghijkl
CRT	3Cut	Sinuana	48,87	ijklm	7,13	opq	0,350	efghijk	281,1	efghijklmn	41,88	ab	3,040	fghij	4,720	efghijklm	7,760	efghijkl
CRT	3Cut	Tai	40,60	mn	8,27	klmnopq	0,400	cdefghij	175,1	lmn	37,82	cdefghij	2,070	hij	3,420	jklm	5,490	jkl
CRT	4Cut	Belloti	57,60	efghi	11,27	bcdefghij	0,320	hijk	248,3	hijklmn	40,05	bcde	2,490	ghij	4,320	ghijklm	6,810	fghijkl
CRT	4Cut	Ropain	72,40	bc	11,87	bcdefghi	0,320	hijk	498,1	bcdef	39,02	bcdefgh	5,470	bc	7,820	bcde	13,280	bcd
CRT	4Cut	Sinuana	61,80	defg	8,67	jklmnopq	0,330	hijk	259,3	ghijklmn	44,20	a	2,850	fghij	4,630	fghijklm	7,490	efghijkl
CRT	4Cut	Tai	56,13	efghij	9,93	ghijklmno	0,310	hijk	292,6	defghijklmn	36,57	efghijk	3,280	efghij	5,080	defghijklm	8,360	defghijkl
CRT	5Cut	Belloti	60,27	defgh	8,20	klmnopq	0,330	ghijk	350,9	cdefghijklmn	36,19	ghijkl	2,950	fghij	4,940	defghijklm	7,890	efghijkl
CRT	5Cut	Ropain	53,40	ghij	10,73	defghijk	0,360	efghijk	491,0	bcdefg	36,51	fghijk	4,610	cdef	6,080	cdefghijk	10,680	bcdefghi
CRT	5Cut	Sinuana	43,13	lm	6,53	qr	0,250	k	211,2	jklmn	38,32	cdefghi	1,850	j	2,620	m	4,460	l
CRT	5Cut	Tai	56,60	efghij	8,80	jklmnopq	0,360	efghijk	400,1	cdefghijkl	35,94	ghijklm	3,890	cdefghi	6,310	cdefghijk	10,200	cdefghij
